# Designing Adjuvant Formulations to Promote Immunogenicity and Protective Efficacy of Leptospira Immunoglobulin-Like Protein A Subunit Vaccine

**DOI:** 10.3389/fcimb.2022.918629

**Published:** 2022-06-16

**Authors:** Teerasit Techawiwattanaboon, Thomas Courant, Livia Brunner, Suwitra Sathean-anan-kun, Pratomporn Krangvichian, Nutta Iadsee, Yaowarin Nakornpakdee, Noppadon Sangjun, Pat Komanee, Nicolas Collin, Kiat Ruxrungtham, Kanitha Patarakul

**Affiliations:** ^1^ Department of Microbiology, Faculty of Medicine, Chulalongkorn University, Bangkok, Thailand; ^2^ Chula Vaccine Research Center (Chula VRC), Center of Excellence in Vaccine Research and Development, Chulalongkorn University, Bangkok, Thailand; ^3^ Vaccine Formulation Institute, Plan-Les-Ouates, Switzerland; ^4^ Medical Microbiology, Interdisciplinary Program, Graduate School, Chulalongkorn University, Bangkok, Thailand; ^5^ Department of Pathobiology, Faculty of Science, Mahidol University, Bangkok, Thailand; ^6^ Laboratory Animal Section, Analysis Division, Armed Force Research Institute of Medical Sciences (AFRIMS), Bangkok, Thailand

**Keywords:** *Leptospira*, immunoglobulin-like protein A, subunit vaccine, adjuvant formulation, neutral liposome, squalene-in-water emulsion, QS21 saponin, QuilA saponin

## Abstract

The leptospirosis burden on humans, especially in high-risk occupational groups and livestock, leads to public health and economic problems. Leptospirosis subunit vaccines have been under development and require further improvement to provide complete protection. Adjuvants can be used to enhance the amplitude, quality, and durability of immune responses. Previously, we demonstrated that LMQ adjuvant (neutral liposomes containing monophosphoryl lipid A (MPL) and *Quillaja saponaria* derived QS21 saponin) promoted protective efficacy of LigAc vaccine against *Leptospira* challenge. To promote immunogenicity and protective efficacy of the subunit vaccines, three alternative adjuvants based on neutral liposomes or squalene-in-water emulsion were evaluated in this study. LQ and LQuil adjuvants combined the neutral liposomes with the QS21 saponin or *Quillaja saponaria* derived QuilA^®^ saponin, respectively. SQuil adjuvant combined a squalene-in-water emulsion with the QuilA^®^ saponin. The immunogenicity and protective efficacy of LigAc (20 µg) formulated with the candidate adjuvants were conducted in golden Syrian hamsters. Hamsters were vaccinated three times at a 2-week interval, followed by a homologous challenge of *L. interrogans* serovar Pomona. The results showed that LigAc combined with LQ, LQuil, or SQuil adjuvants conferred substantial antibody responses and protective efficacy (survival rate, pathological change, and *Leptospira* renal colonization) comparable to LMQ adjuvant. The LigAc+LQ formulation conferred 62.5% survival but was not significantly different from LigAc+LMQ, LigAc+LQuil, and LigAc+SQuil formulations (50% survival). This study highlights the potential of saponin-containing adjuvants LMQ, LQ, LQuil, and SQuil for both human and animal leptospirosis vaccines.

## Introduction

Leptospirosis is the most widespread zoonotic disease with global prevalence. Although it is an emerging public health concern particularly prevalent in tropical and subtropical regions, it is considered a neglected disease (Karpagam and Ganesh, 2020). Incidental infections are common in rural populations, related to the agricultural workforce and living in urban slums (Haake and Levett, 2015). Approximately one million human leptospirosis cases and 60,000 deaths are estimated annually worldwide (Costa et al., 2015), and working-age men have been mostly affected (Torgerson et al., 2015). Various wild and domestic animals are infected with pathogenic leptospires and become reservoir hosts. In addition, leptospirosis causes adverse outcomes in livestock, including infertility, abortion, stillbirth, premature birth, neonatal death, and poor milk production (Ellis, 2015). Therefore, effective vaccines against leptospirosis in humans and animals are necessary. To date, only inactivated whole-cell vaccines (bacterins) are commercially available and licensed for leptospirosis, mainly for animal use (Adler, 2015). Bacterins are approved for human use in a few countries such as France, Cuba, China, and Japan (Adler, 2015), because they fail to activate long-lasting and cross-protective immunity against heterologous serovars and cause undesirable effects. Subunit vaccines have gained increasing attention because they are safer and easier to manipulate to induce broad and long-term immunity than bacterins. However, protein antigens alone might not be immunogenic enough to induce strong immunity, and adjuvants are often required to improve vaccine efficacy (Vogel, 2000).

Currently, one of the most promising candidates for leptospirosis subunit vaccines is the C-terminal region (domain 7-13) of leptospiral immunoglobulin-like protein A (LigAc) (Adler, 2015). The highest survival rate in hamsters was achieved with 100 µg of LigAc combined with Freund’s adjuvant (Silva et al., 2007; Coutinho et al., 2011; Evangelista et al., 2017). However, Freund’s adjuvant is not used in humans or animals because of its harmful side effects resulting from excessive inflammation. LigAc combined with aluminum hydroxide (alhydrogel), a widely authorized adjuvant for human vaccines, showed survival rates of 50%-100%, but sterilizing immunity was not achieved (Faisal et al., 2009; Bacelo et al., 2014; Monaris et al., 2015; Oliveira et al., 2016). Furthermore, certain formulations of LigAc with alhydrogel failed to induce adequate protection (Lucas et al., 2011; Hartwig et al., 2014). LigAc antigen formulated into liposomes or poly-lactic-co-glycolic acid (PLGA) microspheres (Faisal et al., 2009), or formulated with adjuvants including xanthan gum (Bacelo et al., 2014), *Salmonella* flagellin (FliC) (Monaris et al., 2015), and LMQ, which are neutral liposomes containing a monophosphoryl lipid A (MPL) and a purified saponin fraction from *Quillaja saponaria* (QS21) (Techawiwattanaboon et al., 2020), showed promoting survival against lethal challenges. However, none of these adjuvanted vaccine formulations completely prevented *Leptospira* renal colonization (Faisal et al., 2009; Bacelo et al., 2014; Monaris et al., 2015; Techawiwattanaboon et al., 2020). We suggest here that other adjuvants may be investigated for their potential to promote both protection against lethal challenges and renal colonization, especially adjuvants for which cost of goods would be more compatible with animal vaccines.

In our earlier studies, vaccination with 20 µg of LigAc plus LMQ adjuvant protected 60% of hamsters from lethal challenges with *Leptospira* (Techawiwattanaboon et al., 2019; Techawiwattanaboon et al., 2020), which was equivalent to those obtained by 20 µg of LigAc plus Freund’s or alum in other studies (Silva et al., 2007; Faisal et al., 2009). In this study, we evaluated three alternative adjuvants based on neutral liposomes or squalene-in-water emulsion. The LQ and LQuil adjuvants combine neutral liposomes, which act as a carrier for the *Quillaja saponaria* derived QS21 or QuilA^®^ saponins, respectively. The SQuil adjuvant combines a squalene-in-water emulsion with the QuilA^®^ saponin. This evaluation consisted of immunogenicity and protective efficacy studies of LigAc formulated with LQ, LQuil, or SQuil adjuvants in the golden Syrian hamster, an acute lethal model of leptospirosis.

## Materials and Methods

### Bacterial Strains and Culture Conditions

*L. interrogans* serovar Pomona was cultured at 30°C in Ellinghausen-McCullough-Johnson-Harris (EMJH) broth medium, prepared by adding bovine serum albumin (BSA) supplement solution (Zuerner, 2005) to *Leptospira* Medium Base EMJH (BD Difco, Baltimore, MD, USA). *Escherichia coli* were grown at 37°C in Luria-Bertani (LB) medium with the addition of 30 μg/ml kanamycin and 30 μg/ml chloramphenicol when required.

### Preparation of Adjuvant Formulation

LMQ, LQ, LQuil, and SQuil adjuvants were manufactured at the Vaccine Formulation Institute (VFI, Switzerland). The composition of each adjuvant is shown in the **Supplementary Table**. Neutral liposomes, made of DOPC and cholesterol, were prepared by the lipid film method as previously described (Rivera-Hernandez et al., 2020). Briefly, DOPC and cholesterol were dissolved in ethanol and the solvent was evaporated under a vacuum. The lipid film was then rehydrated with Dulbecco’s phosphate-buffered saline (DPBS, pH 7.2) followed by extrusion to yield concentrated neutral liposomes. LMQ was obtained by extemporaneously mixing neutral liposomes with MPL from *Salmonella Minnesota* (Sigma-Aldrich, St. Louis, MO, USA) and QS21 (Desert King International, San Diego, CA, USA). LQ and LQuil were obtained by extemporaneously mixing neutral liposomes with QS21 or QuilA*^®^* (Brenntag, Denmark), respectively. Squalene-in-water emulsion was manufactured as previously described (Ventura et al., 2013), with the addition of cholesterol. SQuil was obtained by extemporaneously mixing squalene-in-water emulsion containing cholesterol with QuilA^®^. Prior to *in vivo* studies, compatibility between each adjuvant and LigAc was confirmed by monitoring the 24-h stability of formulations containing adjuvant and antigen.

### Production of Recombinant LigAc

*L. interrogans* serovar Pomona genome encoding LigAc domain 7-13 (amino acids 631-1225) was amplified using Q5^®^ High-Fidelity DNA polymerase (NEB, MA, USA) with the following primers: LigAc-BamHI-F (5’-ATCGGGATCCCTTACCGTTTCCAACACA-3’) and LigAc-XhoI-R (5’-CGATCTCGAGTGGCTCCGTTTTAATAGA-3’). The amplicon was ligated into pET-24a (Novagen, Darmstadt, Germany) at *Bam*HI and *Xho*I sites. The recombinant plasmids were propagated in *E. coli* DH5α (Novagen) and verified by DNA sequencing (Macrogen Inc., Seoul, South Korea).

The expression of LigAc was induced by 0.5 mM Isopropyl β-D-1-thiogalactopyranoside (IPTG) at 37°C and 200 rpm for 4 h in *E coli* BL21 (DE3) pLysS (Novagen). The bacterial pellets were re-suspended in Tris buffer (50 mM Tris, 200 mM NaCl and 1 mM PMSF, pH 8.0) and disrupted by a high-pressure homogenizer at 4°C. Inclusion body was isolated by centrifugation, washed with washing buffer (0.5% Triton X-100 and 1 M urea in Tris buffer) at 4°C for 3 h, and solubilized in denaturing buffer (6 M urea and 5 mM DTT in Tris buffer) at 4°C overnight. The soluble fraction was loaded onto Bio-Scale™ Mini Nuvia™ IMAC Ni-charged cartridges (Bio-Rad) and serially eluted with denaturing buffer containing two-fold increasing concentrations of imidazole (25 to 400 mM) following the manufacturer’s instructions. The purified LigAc was refolded by multistep dialysis with Tris buffer containing stepwise decreasing concentrations of urea (5 to 0 M). Endotoxin removal was achieved by Pierce™ High-Capacity Endotoxin Removal Spin Columns (Thermo Scientific, Waltham, MA, USA) following the manufacturer’s instructions. The removal was performed and repeated until the amount of endotoxin was less than 0.1 EU/ml.

### Characterization of Recombinant LigAc

The purity and integrity of LigAc were determined by 12% sodium dodecyl sulfate-polyacrylamide gel electrophoresis (SDS-PAGE) under reducing conditions and Western blotting. The polyacrylamide gel was stained with Coomassie brilliant blue R-250 (Bio-Rad). The blotting membrane was blocked with blocking buffer (1% BSA in PBST, composed of PBS and 0.05% Tween 20), and sequentially incubated with 1:5,000 anti-His tag monoclonal antibody (KPL, MD, USA) and 1:5,000 alkaline phosphatase (AP) -conjugated goat anti-mouse antibody (KPL). All incubation steps were performed at room temperature for 1 h followed by three times washing with PBST for 5 min. The immunoreactivity was detected using 1-Step™ NBT/BCIP Substrate Solution (Thermo Scientific). The secondary structure of LigAc was evaluated by Jasco J-815 circular dichroism (CD) spectropolarimeter (Jasco Inc., MD, USA). The amount of endotoxin was measured by Pierce™ LAL Chromogenic Endotoxin Quantitation Kit (Thermo Scientific) following the manufacturer’s instructions.

### Immunogenicity of Adjuvanted LigAc Formulations in Mice

The immunogenicity of LigAc formulated with adjuvants was evaluated in 7/8-week-old C57BL/6J female mice (Charles River) (n = 5 per group) at VFI, Switzerland. Each mouse was immunized twice at a 2-week interval with 5 µg of LigAc formulated with adjuvant in a total volume of 50 µl. For the LigAc+LQ group, animals received 5 µg of QS21 per immunization. For the LigAc+LQuil group, animals received 10 µg of QuilA^®^ per immunization. For the LigAc+SQuil group, animals received 5 µg of QuilA^®^ per immunization. Three weeks after the second immunization, blood samples were collected by cardiac puncture in CO_2_-euthanized animals.

### Immunization and Challenge in Hamsters

Outbred golden Syrian hamsters were obtained from the Animal Unit, Faculty of Medicine, Khon Kaen University, Thailand, which served as the animal resource provider for this study. Female hamsters of 5/6-week-old (n = 5-8 per group) were immunized intramuscularly with vaccine formulations listed in the **Supplementary Table**. Each hamster was immunized three times at 2-week intervals with 20 µg of LigAc adjuvanted in a total volume of 150 µl (75 µl in each hind leg). The optimal ratio of the antigen and each adjuvant was determined and used as follows. For the LigAc+LMQ group, animals received 30 µg of MPL and 30 µg of QS21 per immunization. For the LigAc+LQ group, animals received 15 µg of QS21 per immunization. For the LigAc+LQuil group, animals received 30 µg of QuilA^®^ per immunization. For the LigAc+SQuil group, animals received 15 µg of QuilA^®^ per immunization. One week after the second and third immunizations, blood samples were collected from the lateral saphenous vein. After three immunizations, hamsters were intraperitoneally challenged with 100× LD_50_ (1 × 10^3^ cells) of low-passage (< 5 *in vitro* passages) *L. interrogans* serovar Pomona. The challenged hamsters were weighed and monitored daily for the end-point criteria as previously described (Coutinho et al., 2011). The hamsters that presented any of the end-point criteria or survived up to 4 weeks post-challenge were euthanized with an overdose of isoflurane and exsanguination. Blood and tissue samples were collected for *Leptospira* detection and histopathology analysis.

### Enzyme-Linked Immunosorbent Assay (ELISA)

For the hamster model, each well of Nunc MaxiSorp ELISA Plates (Thermo Fisher Scientific, MA, USA) was coated with LigAc (500 ng), whole-cell lysate of *L. interrogans* serovar Pomona (1 × 10^6^ cells), or BSA (500 ng) in coating buffer (0.1 M Na_2_CO_3_, 0.1 M NaHCO_3_, pH 9.5) at 4°C overnight. The coated wells were blocked with blocking buffer at 37°C for 1 h before incubation with serially diluted hamster sera (five-fold dilution of 1:500 to 1:312,500). The plates were incubated with either 1:5000 horseradish peroxidase (HRP)-labeled goat anti-hamster IgG antibody (KPL), mouse anti-hamster IgG1, IgG2/3, or IgG3 antibodies (Southern Biotech, Birmingham, AL, USA). All incubation steps were performed at 37°C for 1 h followed by three times washing with PBST. The reactivity of sera to antigens was detected using TMB Substrate Set (BioLegend, San Diego, CA, USA) and stopped with 2 M H_2_SO_4_. The absorbance was measured at 450 nm by the Varioskan Flash Spectral Scanning Multimode Reader (Thermo Scientific). Antibody titers were calculated using the midpoint titer from S-curve method with raw OD values (**Supplementary File**). For the mouse model, sera were tested for LigAc-specific antibodies using ELISA plates coated with LigAc (170 ng per well). Mouse serum IgG was detected using a goat anti-mouse IgG coupled to HRP (Southern Biotech).

### Histopathology Determination

The kidney, lung, and liver tissue samples were fixed with 10% neutral buffered formalin, embedded in paraffin, serially sectioned at a thickness of 5 µm, and stained with hematoxylin and eosin. The histopathological examination was performed in a blinded manner using previously described scoring systems (Faisal et al., 2009).

### Detection of Viable Leptospires

Approximately 100 µl of blood samples were inoculated into a semisolid EMJH medium (0.2% agar). About half of the tissue samples were sliced, pulverized by passing through 5 ml syringe, and inoculated into a semisolid EMJH medium. The cultures were serially diluted (10 to 100-fold dilutions) before incubation at 30°C. The presence of viable *Leptospira* was observed weekly under a dark-field microscope for up to 4 weeks.

### Quantitative Real-Time PCR (qPCR)

Total DNA was extracted from approximately 30 mg of kidney tissue by TissueLyser LT (Qiagen) with QIAamp^®^ Fast DNA Tissue Kit (Qiagen) following the manufacturer’s instructions. The qPCR was performed using QuantStudio 5 Real-Time PCR System (Applied Biosystem, CA, USA) and SsoAdvanced™ Universal Probes Supermix (Bio-Rad). The qPCR reaction mixtures consisted of 1× buffer, 100 nM of the probe, and 400 nM of each primer against *lipL32* (Stoddard et al., 2009), and 5 µl of DNA template in a total volume of 20 µl. Leptospiral DNA standard curve was created from 10-fold serially diluted DNA of *L. interrogans* serovar Pomona equivalent to 2 × 10^7^ to 2 × 10^2^ cells/ml.

### Statistical Analysis

All statistical analyses were performed using IBM SPSS Statistics for Windows version 22 and GraphPad Prism 8. The survival curve was plotted using Kaplan-Meier method and the significant difference was calculated by log-rank test. The antibody titers, histopathological scores, viable bacterial detection, and bacterial burden were verified by Mann-Whitney *U*-test.

## Results

### Preparation and Characterization of Recombinant LigAc as a Vaccine Antigen

Recombinant LigAc of *L. interrogans* serovar Pomona was produced in *E. coli* and used as a vaccine antigen to evaluate the efficacy of alternative adjuvants. The recombinant protein was initially expressed in an insoluble form. It was subsequently solubilized in urea and refolded by stepwise dialysis to generate a soluble protein. SDS-PAGE and Western blotting with anti-6× His tag antibody detected approximately 65 kDa band of the LigAc as predicted (**Supplementary Figure S1**). CD spectra illustrated secondary structure motifs of the recombinant LigAc, implying that the refolded conformation of the protein was obtained (**Supplementary Figure S2**). Before use, the protein was prepared at a concentration of 1 mg/ml with a practically undetectable level of endotoxin (less than 0.1 EU/ml).

### Humoral Immune Response Induced by LigAc With Different Adjuvants in Mice

The LigAc-specific IgG levels were measured 3 weeks after the second immunization. LQ, LQuil, and SQuil adjuvants were able to significantly increase the anti-LigAc antibody levels compared to the non-adjuvanted protein (**Supplementary Figure S3**). The liposome-based adjuvants LQ and LQuil induced comparable antibody responses, whereas the antibody responses induced by SQuil were significantly superior despite some variability in antibody titers.

### Humoral Immune Responses Induced by LigAc With Different Adjuvants in Hamsters

**Figure 1:**
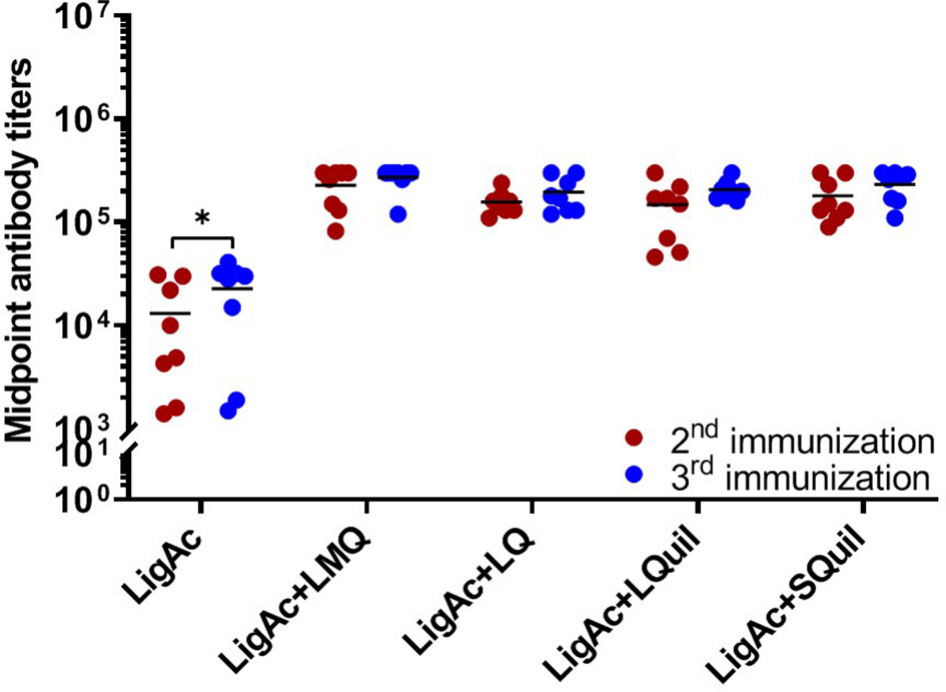
Comparison of LigAc-specific IgG titers after the second and the third immunizations. The antibody titers in the vaccinated hamsters at 1 week after the second and the third immunization were measured by ELISA. Mann–Whitney *U* test was used to compare antibody titers between groups; * represents *p* < 0.05.

The LigAc-specific IgG levels were measured 1 week after the second and the third immunization. No antigen-specific antibody was detected in pre-immune sera and sera from hamsters immunized with only Tris buffer as expected (data not shown). In contrast, high total IgG titers were detected in all hamsters that received LigAc-based immunization (**Figures 1** **–** **3**). There was no significant difference observed between the second and the third immunizations with any adjuvants (**Figure 1**). However, three doses of LigAc without adjuvant induced significantly higher antibody titers than two doses (*p* < 0.01).

**Figure 2:**
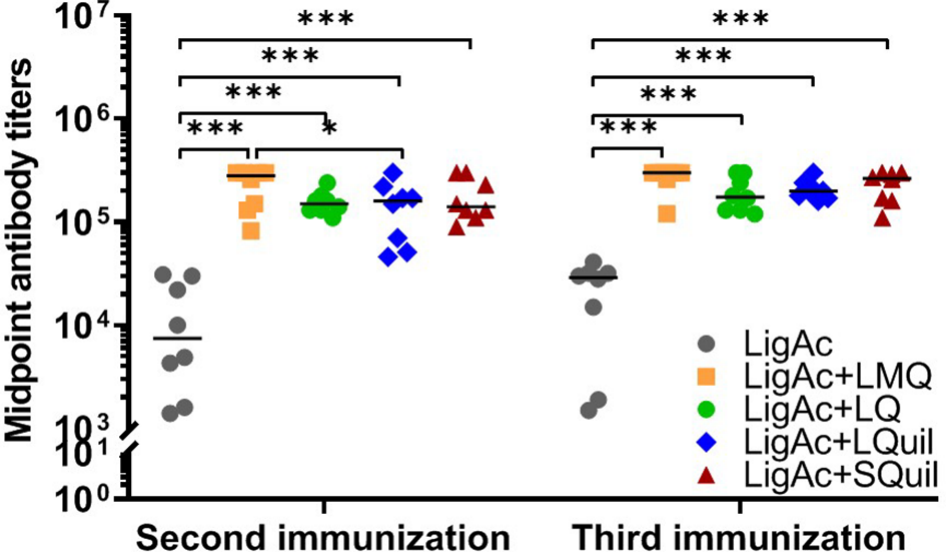
Comparison of LigAc-specific IgG titers among vaccine formulations. The antibody titers in the vaccinated hamsters at 1 week after the second and the third immunization were measured by ELISA. Mann–Whitney *U* test was used to compare antibody titers between groups; * represents *p* < 0.05. and *** represents p < 0.001.

The vaccination with LigAc formulated with each adjuvant induced significantly higher antibody titers than LigAc alone (*p* < 0.001) (**Figure 2**). After the second immunization, LigAc+LMQ vaccine formulation induced comparable antibody levels to LigAc+LQ and LigAc+SQuil formulations but higher than LigAc+LQuil formulation. However, the antibody levels induced by LigAc formulated with any adjuvants were not significantly different after the third immunization.

**Figure 3:**
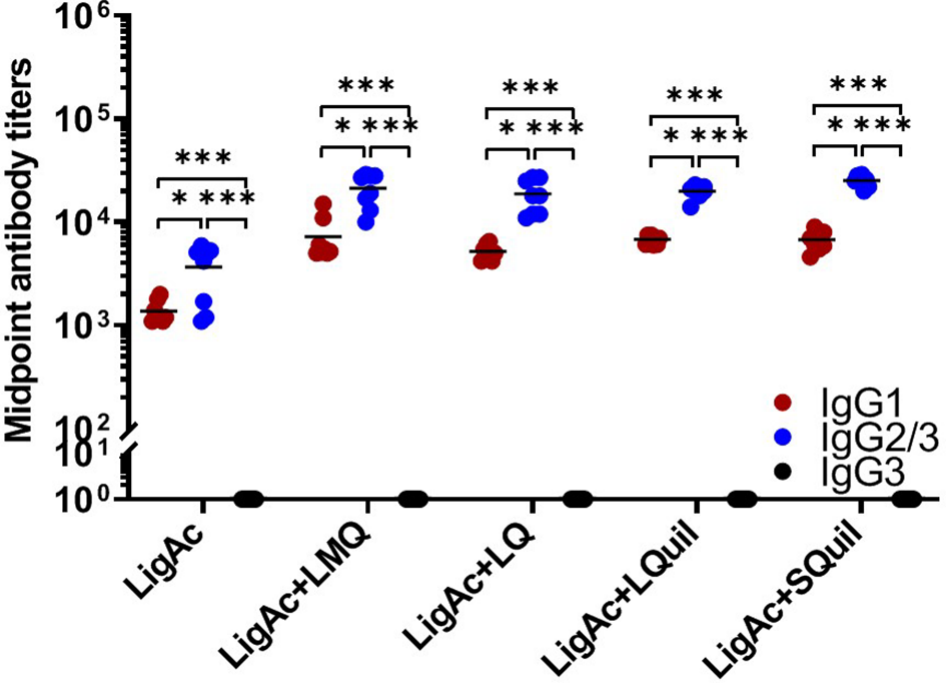
Comparison of LigAc-specific IgG1, IgG2/3, and IgG3 titers. The antibody titers in the vaccinated hamsters at o1 week after the third immunization were measured by ELISA. Mann–Whitney *U* test was used to compare IgG isotype titers between groups; * represents *p* < 0.05. and *** represents p < 0.001.

Furthermore, the IgG subclasses triggered by each LigAc-based vaccine formulation were evaluated in sera after the third immunization. Each formulation induced higher LigAc-specific IgG2/3 titers than IgG1 (*p* < 0.05) and IgG3 (*p* < 0.001) titers (**Figure 3**). Because IgG3 was undetectable, the level of IgG2/3 mainly represented IgG2. The ratios of IgG2 to IgG1 titers were more than one in all hamsters immunized with LigAc combined with any four adjuvants (**Supplementary Figure S4**), suggesting T helper type 1 response. Nevertheless, there was no significant difference in the level of specific IgG2/IgG1 ratio among all tested groups.

### Protective Efficacy of LigAc With Different Adjuvants Against Lethal Challenge

**Figure 4:**
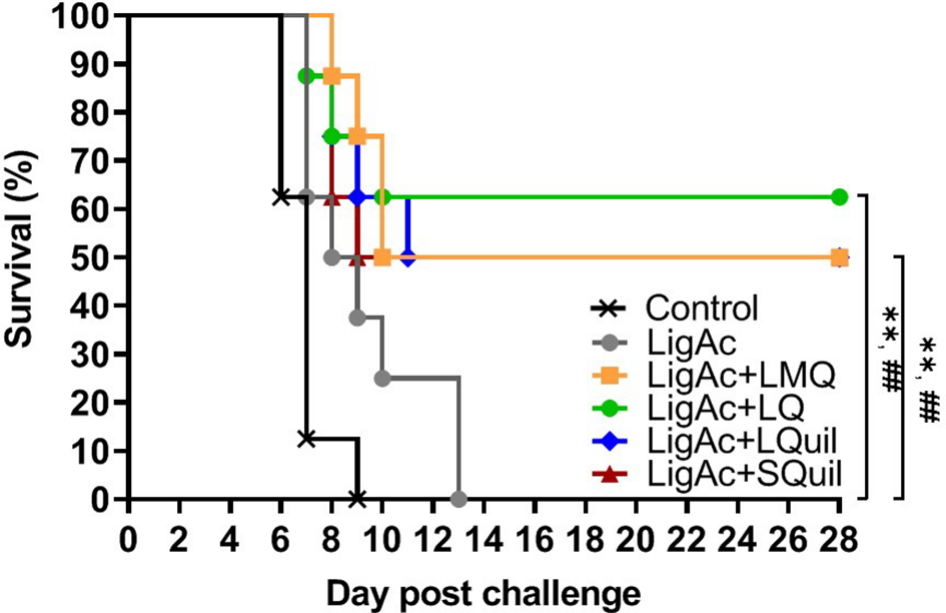
Kaplan–Meier plot of survival rates in vaccinated hamsters (n = 8 per group) following lethal challenge by virulent *Leptospira*. The hamsters were immunized with various vaccine formulations shown in the **Supplementary Table**. Each vaccinated hamster was challenged by 100× LD_50_ of low passage leptospires. The percentage of survival was calculated as the number of survivors/total challenged hamsters ×100. Statistical analysis of survival rates between the negative control group and other vaccinated groups was performed by log-rank test; ** represents *p* < 0.01 (negative control group and vaccinated groups) and ^##^ represents *p* < 0.01 (LigAc alone group and other vaccinated groups).

The survival rate of immunized hamsters following the lethal challenge of virulent *Leptospira* was evaluated to determine the protective efficacy of LigAc formulated with different adjuvants. As expected, all hamsters in the negative control group and those who received LigAc without adjuvant met the end-point criteria within 2 weeks after the challenge (**Figure 4** and **Table 1**). The LigAc formulated with each adjuvant protected at least 50% of hamsters from death, which was significantly higher than the negative control and LigAc alone groups (*p* < 0.01). Although the highest survival rate was 62.5% in the LigAc+LQ formulation group, protection was not significantly different from those of LigAc+LMQ, LigAc+LQuil, and LigAc+SQuil (50% survival) groups. Most non-surviving hamsters showed signs of end-point criteria, including loss of more than 10% body weight. In contrast, all surviving hamsters gained body weight until the end of the experiment.

**Table 1:** Protection conferred by LigAc vaccine formulations.

Group	Protection^a^	Positive culture^b^			Pathology score^c^		
		Kidney	Liver	Lung	Kidney	Liver	Lung
Negative control	0%	NT	NT	NT	NT	NT	NT
LigAc	0%	NT	NT	NT	NT	NT	NT
LigAc+LMQ	50%**, **^##^**	1/4	1/4	0/4	1, 1, 2, 0	0, 0, 1, 0	1, 2, 2, 1
LigAc+LQ	62.5%**, **^##^**	1/5	0/5	1/5	2, 2, 1, 1, 1	1, 1, 0, 0, 1	3, 2, 2, 2, 2
LigAc+LQuil	50%**, **^##^**	0/4	0/4	1/4	2, 1, 1, 3	0, 0, 1, 0	2, 2, 3, 2
LigAc+SQuil	50%**, **^##^**	1/4	0/4	0/4	3, 2, 1, 2	1, 1, 1, 1	2, 2, 2, 2

a The percentage of protection was calculated as the number of surviving/total challenged hamsters × 100. Statistical analysis of survival rate among tested groups was analyzed by log-rank test; ** represents p < 0.01 (negative control group and vaccinated groups) and ^##^ represents p < 0.01 (LigAc alone group and other vaccinated groups).

b Leptospiral culture was performed only in the surviving hamsters. The results show the number of positive culture/total surviving hamsters.

c The pathological scores were determined only in the surviving hamsters. Pulmonary hemorrhage and tubulointerstitial nephritis were graded as 0–3 (none–severe). Liver pathology was graded based on the average number of inflammatory foci in 10 fields at 10× magnification as 0 (none), 1 (1–3), 2 (4–7), or 3 (>7). Mann–Whitney U test was used to compare statistical values of pathological scores among tested groups. NT means not tested.

### Effect of LigAc With Different Adjuvants on a Leptospiral Invasion of Target Organs

**Figure 5:**
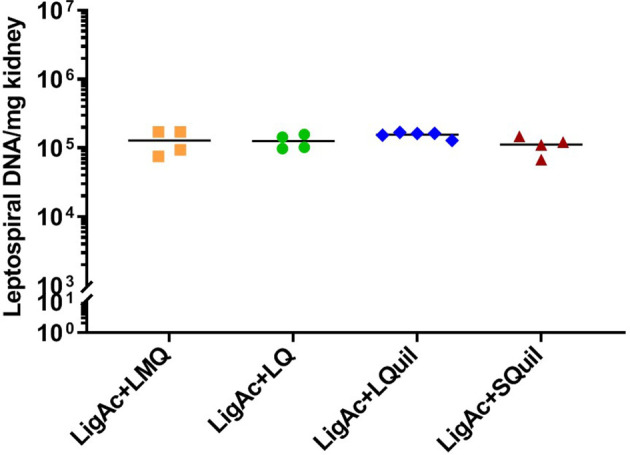
Leptospiral burden in the kidneys of surviving hamsters after challenge. The leptospiral genome was detected by qPCR. The cycle threshold of each sample was compared with leptospiral DNA standard curve to calculate bacterial load, which is expressed as bacterial DNA per milligram of tissue. Mann–Whitney *U* test was used to compare bacterial numbers among vaccinated groups.

Protection against *Leptospira* infection on the target organs of the surviving hamsters was evaluated to determine sterilizing immunity conferred by each vaccine formulation. The culture isolation demonstrated the presence of live leptospires in hamster tissue (**Table 1**). In each vaccinated group, the positive culture detection was from only one surviving hamster, accounting for 20%-25% detection. Comparison among formulations showed no significant difference in the number of *Leptospira*-positive cultures of each organ. All non-surviving hamsters showed viable leptospires only in blood culture, but all other target organs were culture-negative (data not shown). To estimate leptospiral renal colonization, leptospiral DNA in the kidney tissue was determined by qPCR. The leptospiral DNA was detected in all kidney samples of the surviving hamsters. Of these, approximately 1 × 10^5^ cells were detected in each milligram of all kidneys. However, the burden of leptospires was not different among all samples (**Figure 5**), indicating the comparable protection induced by all formulations.

### Prevention of Organ Damage Conferred by LigAc With Different Adjuvants

Histopathological changes were examined in all surviving hamsters to investigate the protection of target organ damage conferred by different vaccine formulations. There was no significant difference in terms of the mean histopathological score of kidneys, livers, and lungs of all survivors (**Table 1**). Tubulointerstitial nephritis indicating renal injury was found in all groups (**Supplementary Figure S5**). No lesion or mild liver inflammation with few inflammatory foci was observed in all survivors (**Supplementary Figure S6**). In addition, mild to moderate lesions with small foci of pulmonary hemorrhage were detected in most surviving hamsters (**Supplementary Figure S7**). Therefore, the vaccination of LigAc formulated with the adjuvants used in this study prevented organ damages comparable to the LigAc+LMQ formulation.

## Discussion

Deleterious effects on humans, especially in high-risk groups and livestock, are the major impact of leptospirosis on public health and economy (Ellis, 2015; Haake and Levett, 2015). Vaccination is the most powerful and cost-effective strategy for preventing infectious diseases. The development of new leptospirosis subunit vaccines currently focuses on overcoming the limited protection and undesirable effects of traditional whole-cell vaccines (Adler, 2015; Felix et al., 2020). However, the immunogenicity of recombinant antigens alone remains unsatisfactory compared with whole-cell vaccines (Gupta et al., 1993). Adjuvants are used in vaccine formulations to improve the immunogenicity of recombinant or subunit antigens and enhance the amplitude, quality, and durability of specific immune responses (Gupta et al., 1993). Previously, we demonstrated that LMQ adjuvant promotes protective efficacy of LigAc antigen but fails to protect against renal colonization (Techawiwattanaboon et al., 2019; Techawiwattanaboon et al., 2020). In this study, we investigated other adjuvants, and our results showed that LigAc combined with the LQ, LQuil, or SQuil adjuvants promoted robust specific antibody responses (**Figures 1**, **2**) and protective efficacy at levels comparable to LMQ (**Figures 4**, **5** and **Table 1**). LQuil and SQuil in particular are adjuvants for which cost of goods are expected to be potentially compatible with animal vaccines.

Liposomes have been extensively used as carriers for immunostimulatory molecules and also as platforms for drug delivery (Nisini et al., 2018). Among different types of liposomes, neutral liposomes have an excellent safety profile (Hernández-Caselles et al., 1993; Nisini et al., 2018). Neutral liposomes have been combined with immunomodulators, such as TLR4 ligands and saponins, to generate new adjuvant formulations (Alving et al., 2012; Deloizy et al., 2017; Rivera-Hernandez et al., 2020; Janitzek et al., 2021).

Saponins are a group of natural products that possess several biological effects, including immunostimulant, antimicrobial, and antitumor activities (Sun et al., 2009). QuilA and QS21 saponins derived from bark extracts from the *Quillaja saponaria* tree have superior adjuvant activity and have been intensively tested as adjuvants in several platforms (Kensil et al., 1991; Sun et al., 2009). QuilA is a heterogeneous mixture of saponins and QS21 is a purified saponin fraction isolated from QuilA using reverse-phase chromatography (RP-HPLC) (Kensil et al., 1991). Saponins exhibit hemolytic activity through the binding of saponin to cholesterol in membrane lipid bilayers, inducing pore formation and membrane leakage (Kensil et al., 1991; Lorent et al., 2014). However, QS21 hemolytic activity can be quenched when it is associated with cholesterol while maintaining adjuvant activity (Beck et al., 2015; Marciani, 2018). QS21 promotes the activation of antigen-presenting cells (Welsby et al., 2016; Marciani, 2018), induces both B and T-cell immune responses (Garçon and Van Mechelen, 2011; Marty-Roix et al., 2016; Marciani, 2018), is currently used in Shingrix^®^ and Mosquirix^®^ vaccines (Lacaille-Dubois, 2019), and is therefore an immunostimulant to be considered for use in new future human vaccines (Zhu and Tuo, 2016). In this study, QS21 was combined with neutral liposomes containing cholesterol to formulate LMQ and LQ. LMQ additionally contained a detoxified derivative of lipid A from *Salmonella* (MPL) which maintains strong immunostimulatory effects (Baldridge et al., 2006). The LigAc combined to LQ induced specific antibody responses and protective efficacy at levels comparable to LMQ (**Figures 1** **–** **5** and **Table 1**). Previously, both formulations were tested with subunit vaccines against group A Streptococcus in mice (Rivera-Hernandez et al., 2020) and induced the same level of protective efficacy. Our results imply that QS21 is the key immunostimulatory component of both formulations associated with neutral liposomes.

QuilA has been reported to induce cellular and humoral immunity (Sun et al., 2009; Naarding et al., 2011; Muller et al., 2019). It is widely used in veterinary but not human vaccines because of its high reactogenicity in humans (Kensil et al., 1991; Sun et al., 2009). In this study, LQuil was generated by mixing QuilA^®^ with neutral liposomes to quench its lytic activity. Despite the difference in the saponin content of LQuil and LQ, they both induced comparable LigAc-specific antibody levels and protective immunity (**Figures 1** **–** **5** and **Table 1**). SQuil is the combination of QuilA^®^ and a squalene-based oil-in-water emulsion. Squalene-in-water emulsions are known to induce innate immune cell infiltration to the vaccination sites as well as enhance antigen uptake and presentation to adaptive immune cells (O’Hagan et al., 2012). In this study, SQuil had the same efficacy as LQuil in that SQuil protected hamsters from the lethal challenge at a similar level to that of LMQ and LQ (**Figures 4**, **5** and **Table 1**). Our results highlight the potential of saponin-containing adjuvants LMQ, LQ, LQuil, and SQuil for leptospirosis vaccines.

Several studies have indicated that protection against leptospirosis is associated with a mixed Th1/Th2 immunity (Faisal et al., 2009; Oliveira et al., 2016; da Cunha et al., 2019; Techawiwattanaboon et al., 2019). However, the predominant IgG isotypes or Th immune responses correlating with protection against leptospirosis are not clearly understood. The recombinant Lig chimeric antigen formulated with Montanide and alhydrogel adjuvants induced different types of humoral immune responses (Th1-biased and mixed Th1/Th2 responses, respectively) (da Cunha et al., 2019), but both formulations conferred 100% protection. In another study, LigAc adjuvanted with liposomes and PLGA microspheres induced Th2-biased and balanced Th1/Th2 responses, respectively, and conferred a comparable rate of protection (Faisal et al., 2009). In contrast, despite inducing similar types of immune responses, LigAc adjuvanted with alhydrogel conferred significantly higher protection than LigAc adjuvanted with carboxyl multi-walled carbon nanotubes (COOH-MWCNTs) that failed to induce protection (Oliveira et al., 2016). In this study, all adjuvants induced higher IgG2 than IgG1, indicating Th1 responses (**Figure 3**). Therefore, all adjuvants improved the immunogenicity and protective efficacy of LigAc by inducing the specific IgG antibody responses.

This study has some limitations. First, antigen-specific T cell type response was not directly determined because the major protective immunity against leptospiral infection is presumably humoral immune response (Adler and Faine, 1977; Chassin et al., 2009). The effect of each vaccine formulation on T helper cell response should be further investigated. However, the IgG isotype was determined to imply T helper response in this study. Second, the challenge of *Leptospira* was intraperitoneal route, which is not the natural route of infection. Third, chronic renal colonization was not determined by long-term detection of urinary shedding. This study only detected leptospires in the kidneys by culture and qPCR of leptospiral DNA. Fourth, the immunization control was not performed. Although the immunization group without leptospiral challenge was not included, no hamsters showed clinical signs of illness or weight loss before the challenge. In addition, the pathological changes of target organs in hamsters that reached the end-point criteria in this study were compatible with severe and lethal leptospirosis similar to previous reports in hamsters (Gomes-Solecki et al., 2017 and Haake, 2006). Therefore, pathological changes of target organs after challenge should be a result of *Leptospira* infection rather than the effects of vaccine formulations.

Ideally, leptospirosis vaccines should be safe and effective, induce long-term immunity, be easy to administer, stable, and be low-cost. Our results indicated the capability of four adjuvants, LMQ, LQ, LQuil, and SQuil, to enhance immunogenicity and protective efficacy of LigAc antigen against lethal challenges in hamsters (**Figures 1** **–** **5** and **Table 1**). However, based on the cost and complexity of vaccine production, LQ, LQuil, and SQuil may be more attractive alternatives to the previously studied LMQ, especially for animal vaccines. In addition, two doses of all four vaccine formulations may be sufficient to confer protection as no significant differences in LigAc-specific antibody levels were observed after the second and third immunizations. Subunit vaccines using only LigAc as an antigen might not be sufficient to confer complete protection against leptospiral infection. We are searching for additional vaccine antigen candidates to promote enhanced vaccine efficacy and better protection against *Leptospira* infection. In this study, we have identified several adjuvants which could be used with new subunit vaccines for human and animal leptospirosis.

## Data Availability Statement

The original contributions presented in the study are included in the article/**Supplementary Material**. Further inquiries can be directed to the corresponding author.

## Ethics Statement

The animal study was reviewed and approved by the Swiss Federal Animal Protection Act and the Thai National Animals for Scientific Purposes Act, BE 2558 (AD 2015) under licenses issued by the Institute for Animals for Scientific Purpose Development and National Research Council of Thailand. The protocols for hamsters were approved by the Institutional Animal Care and Use Committee (IACUC) of the Armed Forces Research Institute of Medical Sciences (AFRIMS), Thailand (approval no. ARAC 1/62).

## Author Contributions

TT: Conceptualization, Methodology, Investigation, Formal analysis, Project administration, Writing-original draft preparation, Writing – review & editing. TC: Conceptualization, Methodology, Investigation, Formal analysis, Writing-original draft preparation, Writing – review & editing. SS, PKr, NI, YN, and PKo: Methodology, Investigation, Formal analysis. LB and NS: Conceptualization, Methodology, Investigation, Formal analysis. NC: Conceptualization, Supervision, Funding acquisition, Writing – review & editing. KR: Conceptualization, Supervision. KP: Conceptualization, Project administration, Supervision, Funding acquisition, Writing – review & editing. All authors contributed to the article and approved the submitted version.

## Funding

This research project was supported by the Thailand Center of Excellence for Life Sciences (grant “Southeast Asia-Europe Join Funding Scheme for Research and Innovation), Chulalongkorn University (grant no. GB-B_61_056_30_14 and GB-CU-61-12-30-02”), and the Ratchadaphiseksomphot Fund of Faculty of Medicine, Chulalongkorn University (grant no. RA-MF-41/64), the Second Century Fund (C2F), Chulalongkorn University (grant “Post-doctoral fellowship for Teerasit Techawiwattanaboon”).

## Conflict of Interest

The authors declare that the research was conducted in the absence of any commercial or financial relationships that could be construed as a potential conflict of interest.

## Publisher’s Note

All claims expressed in this article are solely those of the authors and do not necessarily represent those of their affiliated organizations, or those of the publisher, the editors and the reviewers. Any product that may be evaluated in this article, or claim that may be made by its manufacturer, is not guaranteed or endorsed by the publisher.
