# Correction: Designing adjuvant formulations to promote immunogenicity and protective efficacy of *Leptospira* immunoglobulin-like protein a subunit vaccine

**DOI:** 10.3389/fcimb.2025.1656235

**Published:** 2025-08-18

**Authors:** Teerasit Techawiwattanaboon, Thomas Courant, Livia Brunner, Suwitra Sathean-anan-kun, Pratomporn Krangvichian, Nutta Iadsee, Yaowarin Nakornpakdee, Noppadon Sangjun, Pat Komanee, Nicolas Collin, Kiat Ruxrungtham, Kanitha Patarakul

**Affiliations:** ^1^ Department of Microbiology, Faculty of Medicine, Chulalongkorn University, Bangkok, Thailand; ^2^ Chula Vaccine Research Center (Chula VRC), Center of Excellence in Vaccine Research and Development, Chulalongkorn University, Bangkok, Thailand; ^3^ Vaccine Formulation Institute, Plan-Les-Ouates, Switzerland; ^4^ Medical Microbiology, Interdisciplinary Program, Graduate School, Chulalongkorn University, Bangkok, Thailand; ^5^ Department of Pathobiology, Faculty of Science, Mahidol University, Bangkok, Thailand; ^6^ Laboratory Animal Section, Analysis Division, Armed Force Research Institute of Medical Sciences (AFRIMS), Bangkok, Thailand

**Keywords:** *Leptospira*, immunoglobulin-like protein A, subunit vaccine, adjuvant formulation, neutral liposome, squalene-in-water emulsion, QS21 saponin, QuilA saponin

The animal facility was misidentified in the **Materials and Methods** section.

A correction has been made to the section **Materials and Methods**, subsection *Immunization and Challenge in Hamsters*, paragraph 1:

The sentence originally read:

“Outbred golden Syrian hamsters were purchased from the North-East Animal Laboratory Center, Khon Kaen University, Thailand.”

It has been corrected to:

“Outbred golden Syrian hamsters were obtained from the Animal Unit, Faculty of Medicine, Khon Kaen University, Thailand, which served as the animal resource provider for this study.”

The original version of this article has been updated.

