# Supplementary material for: Designing Adjuvant Formulations to Promote Immunogenicity and Protective Efficacy of Leptospira Immunoglobulin-Like Protein A Subunit Vaccine
Source: Front Cell Infect Microbiol. 2022 Jun 16;12:918629. doi: 10.3389/fcimb.2022.918629 (PMC9243587; doi:10.3389/fcimb.2022.918629)
Supplement: Supplementary file 1 [file DataSheet1.docx]

**Characterization of recombinant LigAc as a vaccine antigen.**


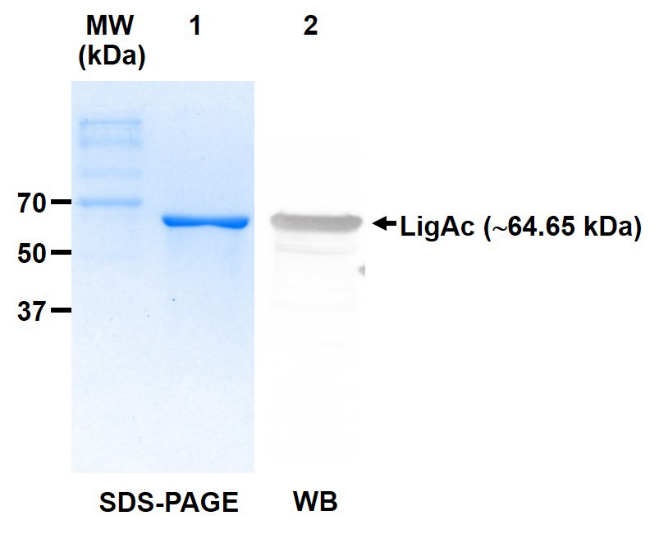


**Supplementary Figure 1** Analysis of the purified recombinant LigAc by SDS-PAGE and Western blotting (WB). Lane M = Precision Plus Protein™ All Blue Prestained Protein Standards (Bio-Rad), lane 1 = LigAc stained with Coomassie Brilliant Blue R-250, and lane 2 = LigAc detected by WB with anti-6× His tag antibody.

**Characterization of recombinant LigAc as a vaccine antigen (continue).**

**Supplementary Figure 2** Analysis of the purified recombinant LigAc by circular dichroism (CD) analysis. The CD spectra were measured by a JASCO J-815-150S spectropolarimeter and analyzed with CDPro software. The CD spectrum represents an average of five spectra from 190 to 260nm.

**Humoral immune response induced by LigAc with different adjuvants in mice.**

**
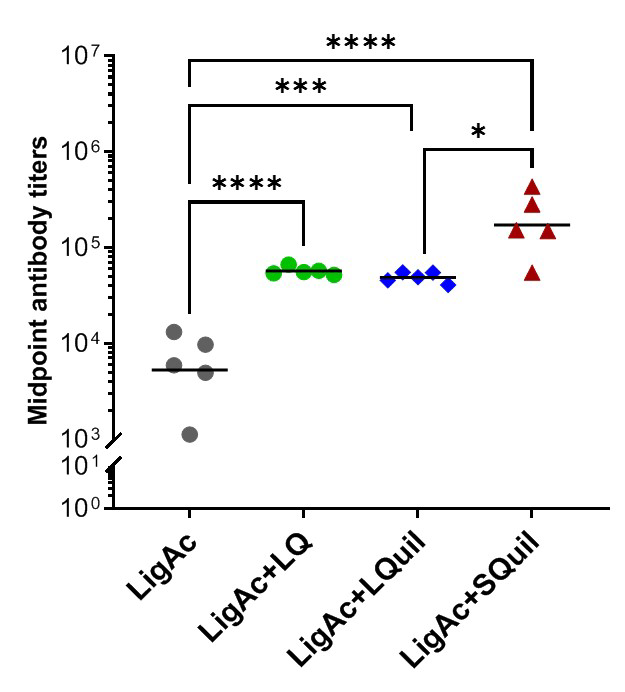
**

**Supplementary Figure 3** LigAc-specific total IgG levels in the mice vaccinated with different adjuvanted LigAc formulations. Antibody titers in sera collected three weeks after the second immunization were measured by ELISA. One-way ANOVA with correction for multiple comparisons using Tukey Test was used to compare antibody titers between groups; * represents *p* < 0.05, *** represents *p* < 0.001 and **** represents *p* < 0.0001.

**The ratio of LigAc-specific IgG2/IgG1 antibody titers** **in the vaccinated hamsters.**

**Supplementary Figure 4** LigAc-specific IgG subclass levels in the vaccinated hamsters. The ratio of LigAc-specific IgG2/IgG1 antibody titers. Mann–Whitney *U* test was used to compare IgG isotype titers between groups; * represents *p* < 0.05, and ** represents *p* < 0.01.

**Tubulointerstitial nephritis indicating renal injury in the vaccinated hamsters**


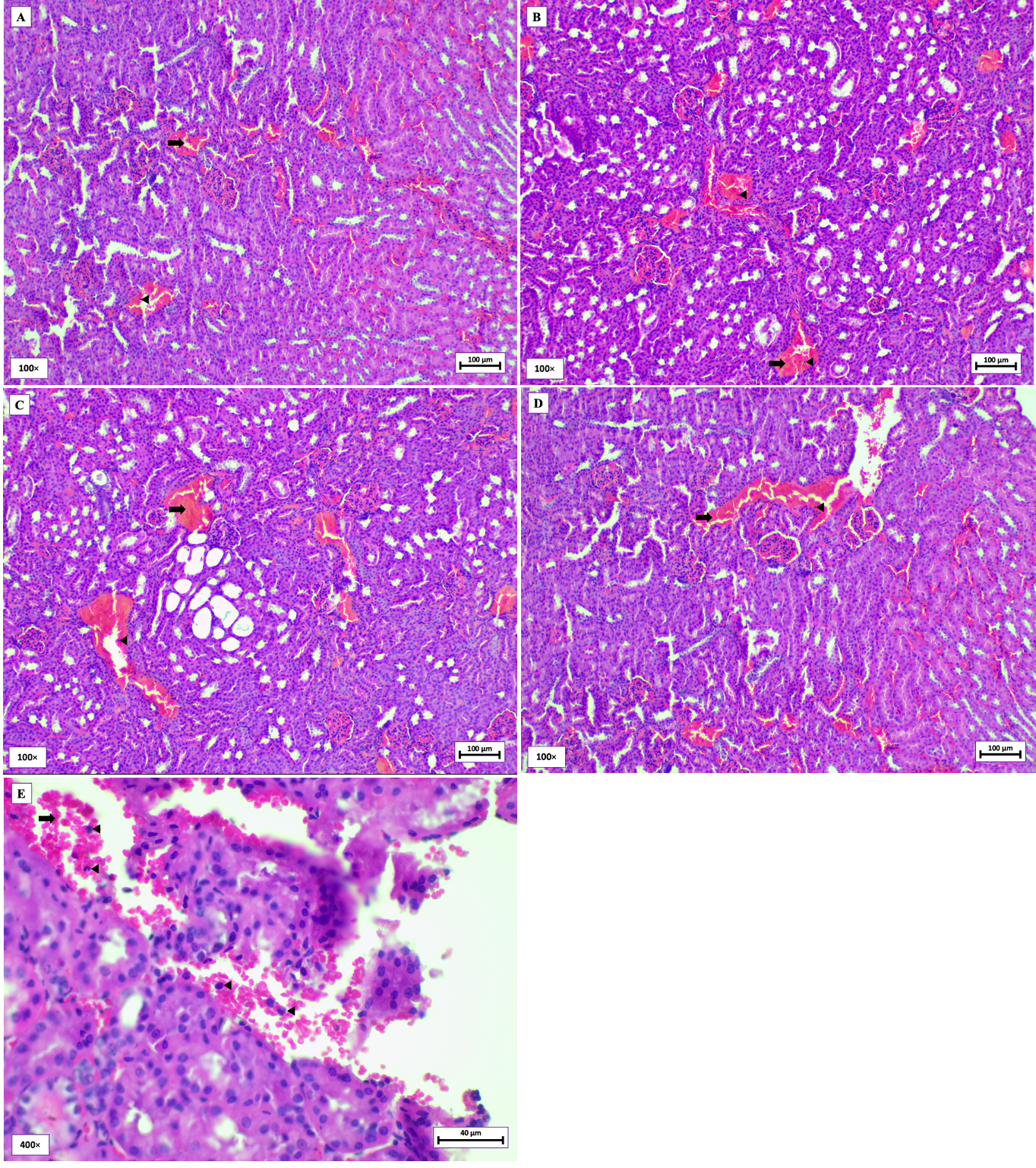


**Supplementary Figure 5** Kidney histopathology showing tubulointerstitial nephritis from hamsters vaccinated with various vaccine formulations (**TABLE 1**). Hemorrhage (black arrows) and inflammatory immune cell infiltration (black arrowheads) were visualized under light microscope. (A) LigAc+LMQ; (B) LigAc+LQ; (C) LigAc+LQuil; (D) LigAc+Squil. Black bar represents 100 μm (H&E; magnification 100×).; (E) Tubules are separated by inflammation and several inflammatory cells are invading the tubules leading to tubular basement membrane disruption. Black bar represents 40 μm (H&E; magnification 400×).

**No lesion or mild liver inflammation with few inflammatory foci in the vaccinated hamsters**


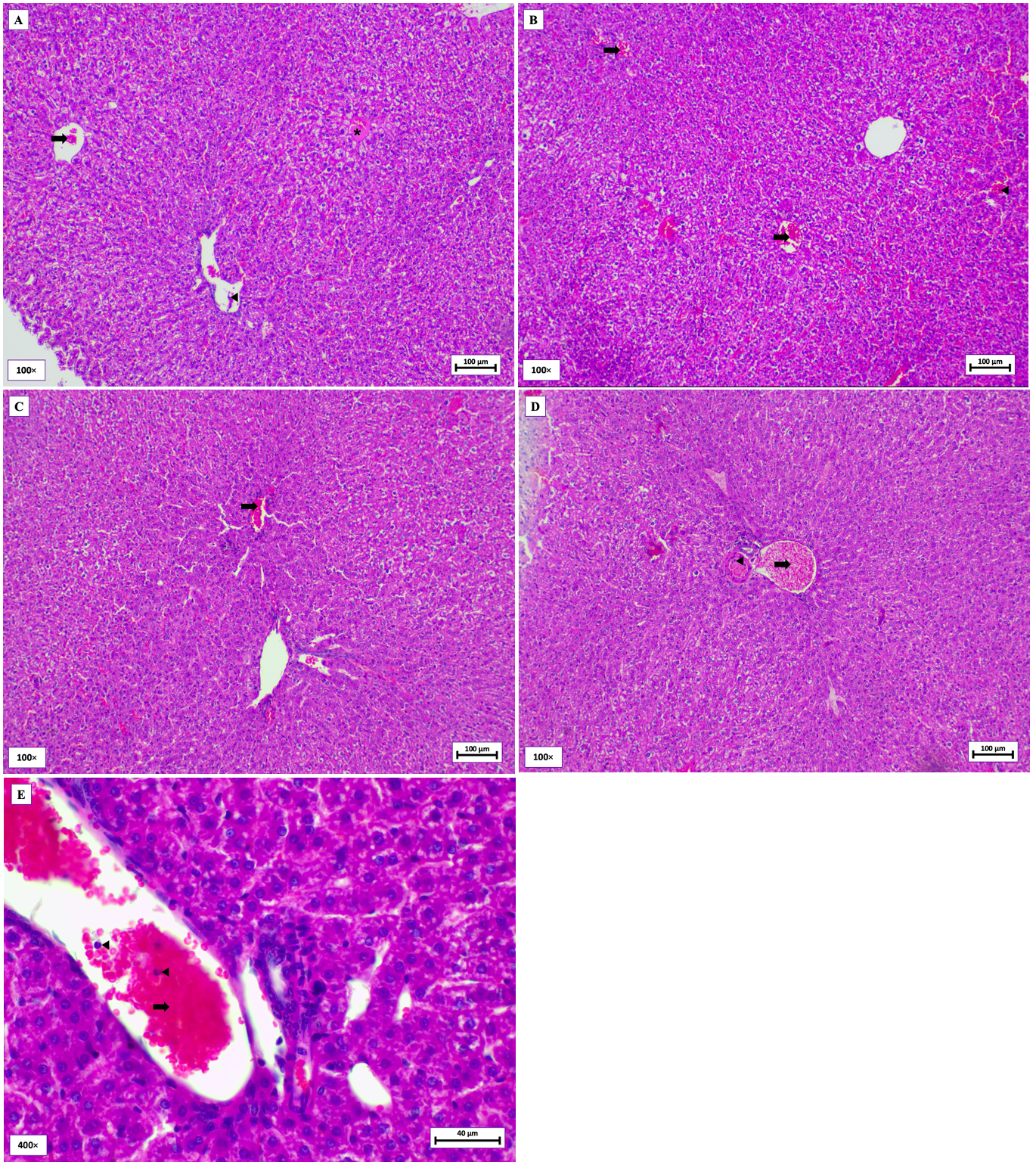


**Supplementary Figure 6** Liver histopathology showing mild inflammation in hamsters vaccinated with various vaccine formulations (**TABLE 1**). Congestion or hemorrhage (black arrows), edema (asterisk), and inflammatory immune cell infiltration (black arrowheads) were visualized under light microscope. (A) LigAc+LMQ; (B) LigAc+LQ; (C) LigAc+LQuil; (D) LigAc+SQuil. Black bar represents 100 μm (H&E; magnification 100×).; (E) Mild congestion with normal histological appearance of the hepatocytes and inflammatory cell infiltration. Black bar represents 40 μm (H&E; magnification 400×).

**Mild to moderate lesions with small foci of pulmonary hemorrhage in the vaccinated hamsters**

**
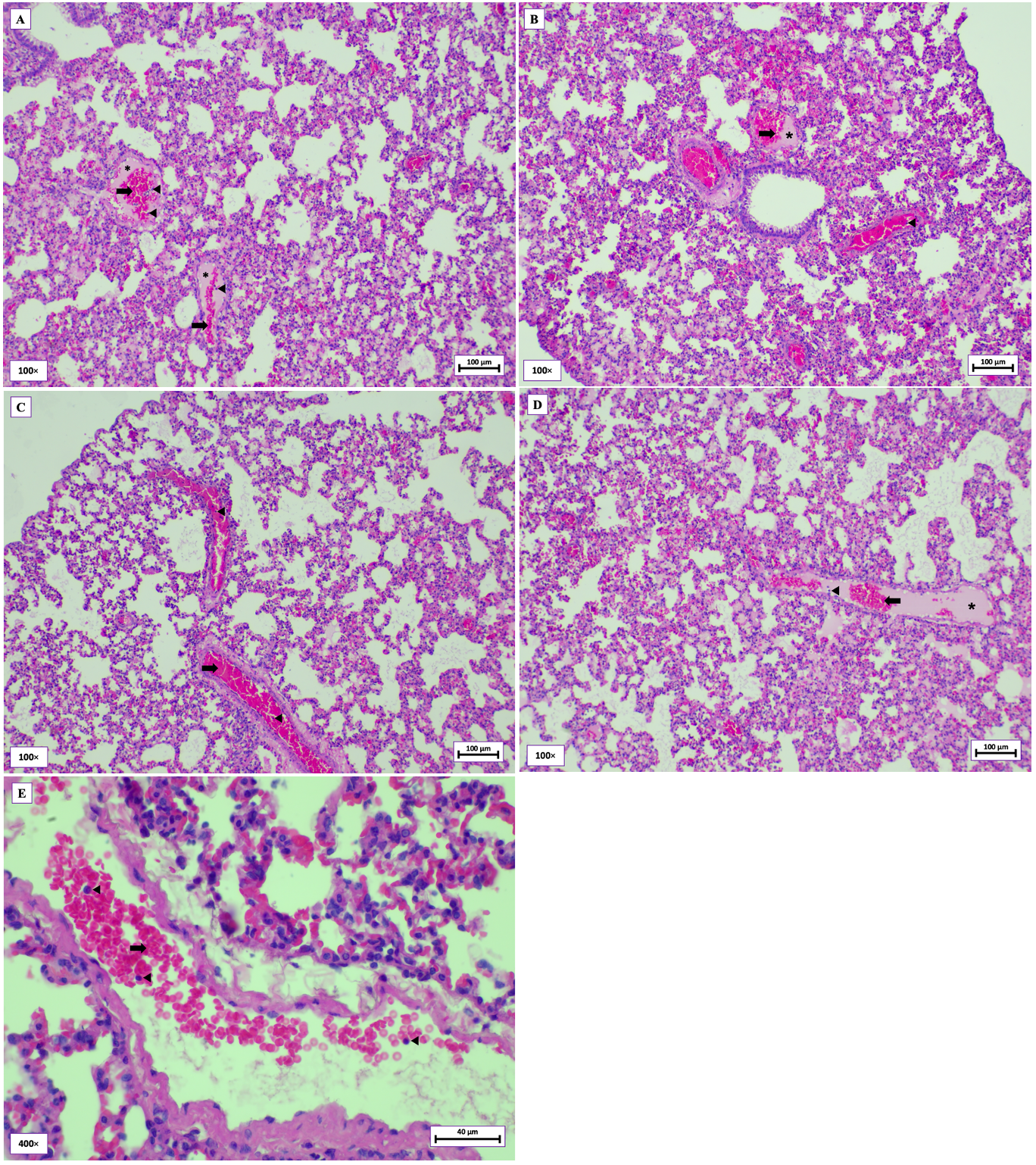
**

**Supplementary Figure 7** Lung histopathology showing small foci from hamsters vaccinated with various vaccine formulations (**TABLE 1**). Congestion or hemorrhage (black arrows), alveolar edema (asterisk), and inflammatory immune cell infiltration (black arrowheads) were visualized under light microscope. (A) LigAc+LMQ; (B) LigAc+LQ; (C) LigAc+LQuil; (D) LigAc+SQuil. Black bar represents 100 μm (H&E; magnification 100×).; (E) Alveolar spaces filled with blood and some inflammatory cells. Black bar represents 40 μm (H&E; magnification 400×).
