# Supplementary material for: Designing Adjuvant Formulations to Promote Immunogenicity and Protective Efficacy of Leptospira Immunoglobulin-Like Protein A Subunit Vaccine
Source: Front Cell Infect Microbiol. 2022 Jun 16;12:918629. doi: 10.3389/fcimb.2022.918629 (PMC9243587; doi:10.3389/fcimb.2022.918629)
Supplement: Supplementary file 2 [file DataSheet2.docx]

**Supplementary table** Vaccine formulations.

| Group | Antigen | Adjuvant composition |
| --- | --- | --- |
| Negative control | Tris buffer | - |
| LigAc alone | LigAc | - |
| LigAc+LMQ | LigAc | Neutral liposomes + MPL + QS21 |
| LigAc+LQ | LigAc | Neutral liposomes + QS21 |
| LigAc+LQuil | LigAc | Neutral liposomes + QuilA^®^ |
| LigAc+SQuil | LigAc | Squalene-in-water emulsion containing cholesterol + QuilA^®^ |
